# Congenital Insensitivity to Pain With Anhidrosis: First Reported Case in Nepal

**DOI:** 10.1002/ccr3.70697

**Published:** 2025-07-28

**Authors:** Sobin Pant, Dibij Adhikari, Prakash Pandey, Binit Bajracharya, Rizuna Sharma

**Affiliations:** ^1^ Maharajgunj Medical Campus, Institute of Medicine Tribhuvan University Kathmandu Nepal; ^2^ Department of Pediatrics Tribhuvan University Teaching Hospital Kathmandu Nepal

**Keywords:** anhidrosis, congenital insensitivity to pain with anhidrosis, hereditary sensory and autonomic neuropathy type IV, pain insensitivity, resource‐limited settings, self‐mutilation

## Abstract

Congenital insensitivity to pain with anhidrosis is a rare autosomal recessive disorder characterized by anhidrosis, self‐mutilation, and insensitivity to pain and temperature. While genetic testing confirms the diagnosis, it is not always feasible, making clinical recognition crucial in resource‐limited settings. Early diagnosis and a multidisciplinary approach help prevent complications like severe injuries, infections, and hyperpyrexia.

## 1 Introduction

Congenital insensitivity to pain with anhidrosis (CIPA), also known as hereditary sensory and autonomic neuropathy (HSAN) type IV, is a rare autosomal recessive disorder. It is characterized by profound and diffuse insensitivity to pain and temperature [1]. Diminished pain perception results in an impaired ability to protect oneself from external or self‐inflicted injuries. Consequently, self‐mutilation is a common and severe complication, frequently resulting in significant injuries, including the loss of digits or other body parts [1, 2]. Fractures are also common and slow to heal, and large weight‐bearing joints are susceptible to repeated trauma and frequently go on to develop Charcot joints and osteomyelitis [1, 3].

The cardinal feature of CIPA is the presence of widespread anhidrosis [1]. The absence of sweating causes episodic fevers and extreme hyperpyrexia due to the inability to regulate body temperature. Recurrent episodic fevers are usually the earliest sign of the disorder and can cause febrile convulsions, especially when in a hot environment [4]. Absent sweating also causes the skin to become thick and callused, with lichenification of palms, dystrophic nails, and areas of hypotrichosis on the scalp [1, 5]. Other than the characteristic anhidrosis, other autonomic disturbances are mild to absent [6].

Sensory loss is observed only in pain and temperature. Touch, vibration, and position senses are preserved. Peripheral motor and cranial nerve function are normal. Deep tendon reflexes are normal, as well as superficial abdominal and cremasteric reflexes [7]. Corneal reflexes are inconsistently present. Blinking and lacrimation are normal [8]. Corneal ulcerations are uncommon but may occur as a result of corneal hypoesthesia and can develop into corneal opacities [9].

CIPA is an exceedingly rare condition, with only a limited number of cases reported worldwide. The estimated prevalence is approximately 1 in 125 million births [10]. To the best of our knowledge, this is the first case report of CIPA from Nepal. We present the case of a 5‐year‐old male child, highlighting its clinical features and the challenges in diagnosis in a resource‐limited setting.

## 2 Case History/Examination

The patient, a 5‐year‐old male, presented with repetitive self‐injurious behaviors like excessive biting of his fingertips, lips, and tongue, first observed at around 6 months of age. These behaviors worsened during periods of discomfort and irritability, resulting in tissue damage, including fingertip mutilation and tissue loss around the lips. At approximately 1 year of age, his parents noticed a loss of pain sensitivity, as the child showed no response to accidental exposure to warm water, pinpricks, or burns from fire. Additionally, the child had a history of recurrent febrile illnesses with a few episodes of febrile seizures, first observed around 6 months of age. Some of these febrile seizures were accompanied by loss of consciousness, body stiffening, and abnormal movements.

The child was born at term via spontaneous vaginal delivery with a normal birth weight. However, he did not cry at birth and required neonatal intensive care for the first 3 days of life. He was bottle‐fed until 4 months of age, after which food supplements were introduced. The child showed severe developmental delays across all four domains (Table 1), with a developmental quotient of 18.8%, indicating profound developmental delay.

**TABLE 1:** Developmental milestones across key domains.

Domain of development	Activity	Developmental age (months)
Gross motor	Stand with support but unable to walk	~9
Fine motor	Immature pincer grasps present but without scribbling	~9
Language	Can produce bisyllabic sounds but can't speak words	~9
Social	Comes when called but doesn't copy parents in tasks	~12

**FIGURE 1:**
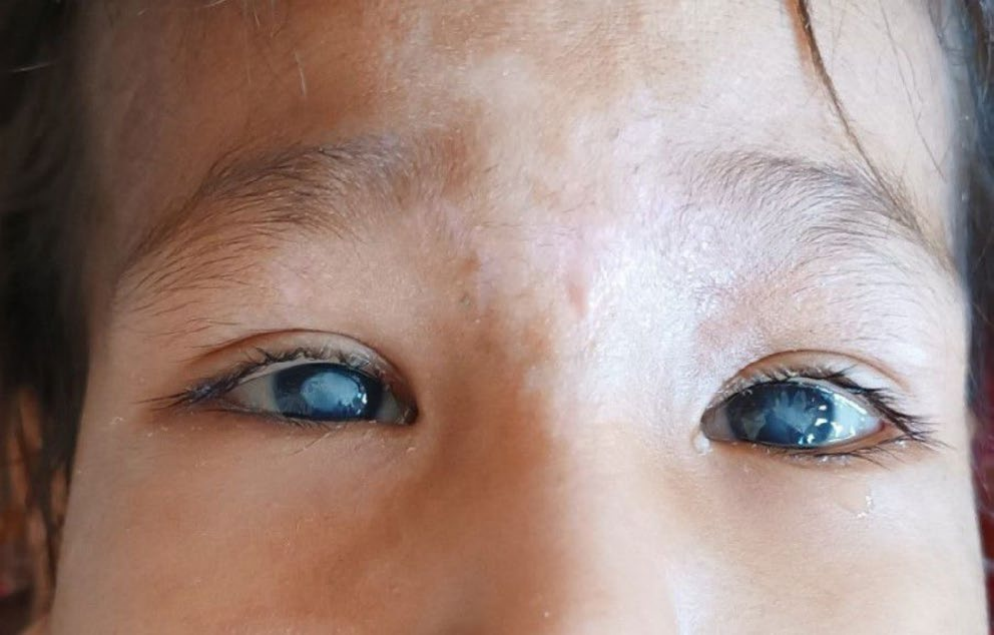
The image shows bilateral corneal opacities, which may be due to recurrent corneal injuries resulting from impaired pain perception.

**FIGURE 2:**
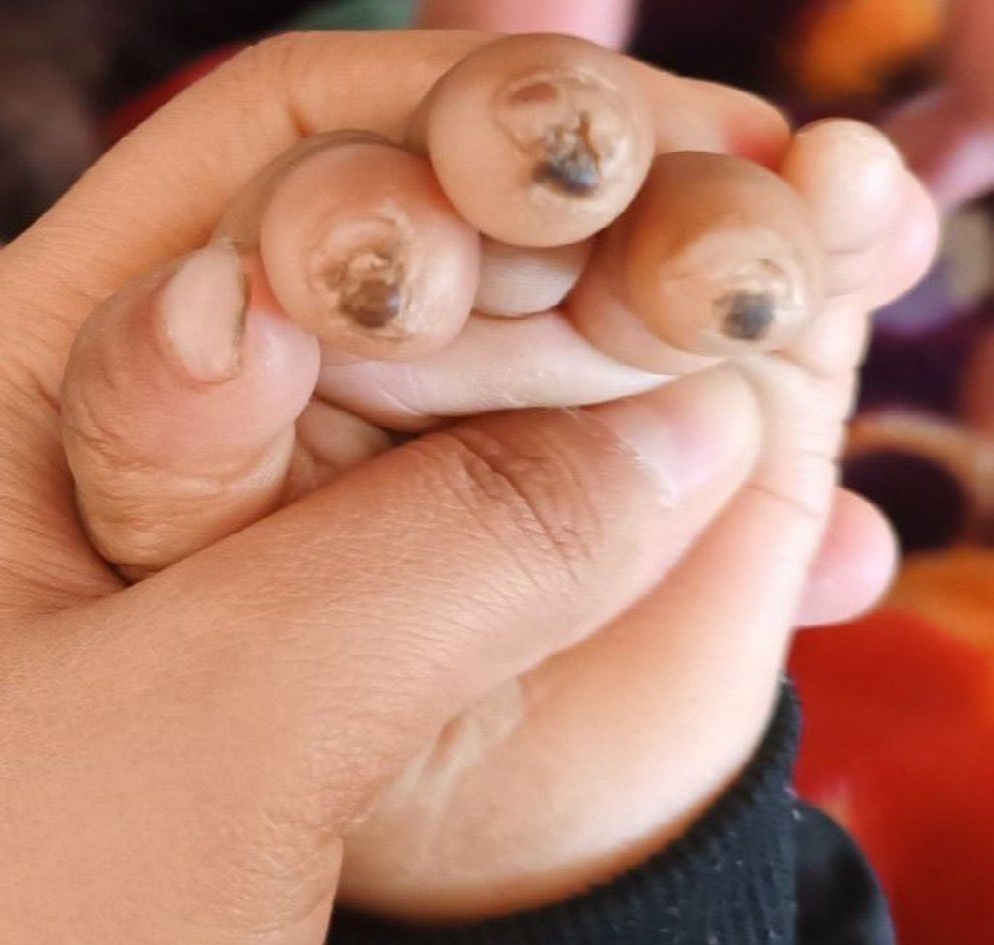
The image shows ulcerative lesions and tissue loss of fingertips due to repetitive self‐biting.

**FIGURE 3:**
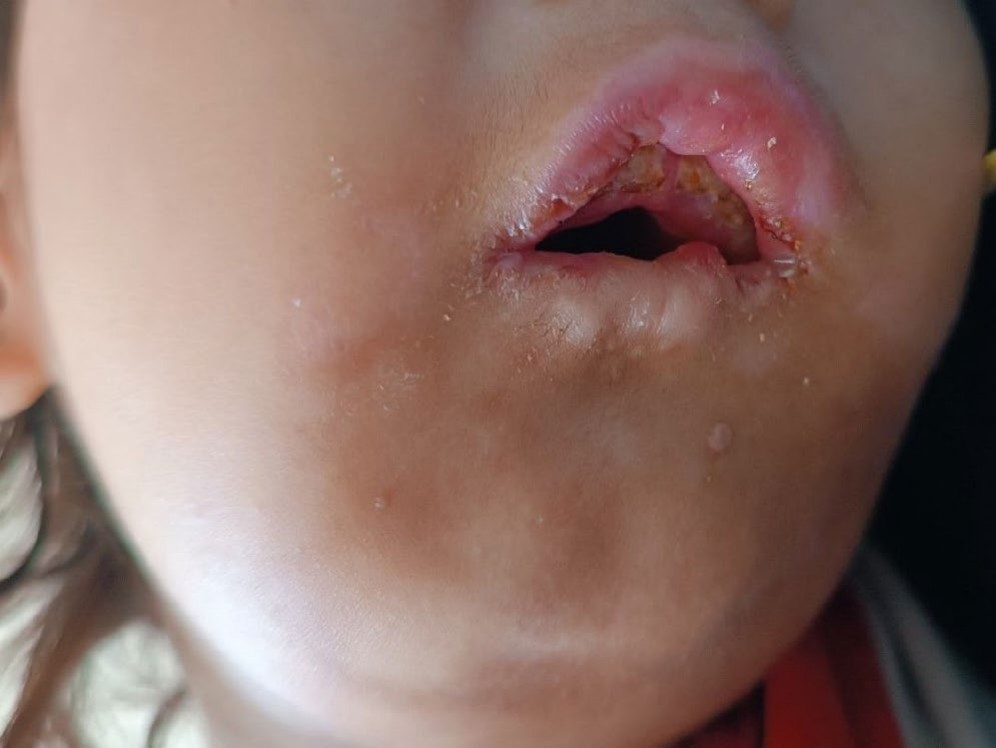
The image shows self‐inflicted injuries, including ulcerations, tissue loss in the lips, and gingival swelling. The upper teeth are underdeveloped.

On physical examination, the child exhibited moderate pallor, retrognathia with a deformed mandibular alveolar ridge, bilateral corneal opacities (Figure 1), and patchy alopecia in the anterior scalp. Ulcerative lesions were noted on the tips of most of his fingers (Figure 2), along with diffuse swelling of distal phalanges and distal interphalangeal joints. There was diffuse gingival swelling, multiple ulcerative lesions, and tissue loss in both lips. The teeth in the upper jaw were underdeveloped, while there were no teeth in the lower jaw (Figure 3). The skin on the palms and soles was thick and dry. No joint or skeletal abnormalities were noted. Anthropometric measurements revealed that the child was severely underweight and stunted, with height, weight, and age measurements all below 3 standard deviations.

On neurological examination, the child exhibited normal muscle bulk, tone, and power in all four limbs. Deep tendon reflexes were symmetrical and preserved, with no signs of upper motor neuron involvement.

The child demonstrated normal light touch sensation, as evidenced by orienting responses to gentle tactile stimuli (using cotton wisp) across the limbs and trunk. However, pain and temperature sensations were impaired in the trunk, extremities, and face. The child did not show any response to pinprick or temperature stimuli. Vibration and proprioception responses could not be reliably assessed due to the child's developmental age and uncooperativeness during the examination.

Cranial nerve assessment was unremarkable, except for an absent corneal reflex. Pupils were equal and reactive to light and accommodation. There were no abnormalities in extraocular movements, facial symmetry, or gag reflex. No ptosis was observed.

Autonomic features were evident with generalized anhidrosis, confirmed through history and clinical observation, including the absence of sweating during febrile episodes. The child also presented with recurrent high‐grade fevers, suggestive of impaired thermoregulation. Tear production was normal. Cardiovascular functions, including heart rate and blood pressure, were stable, with no significant changes with postural changes.

## 3 Investigations and Treatment

The blood investigation revealed that his hemoglobin level was 8.2 mg/dL, his platelet level was 628,000 per cubic millimeter, and the total leucocyte count was within normal limits. Serum uric acid was also within normal limits. The ferritin level was low, while the total iron‐binding capacity was raised, suggesting that the child had iron deficiency anemia. The nerve conduction study showed no specific changes to draw relevant conclusions. Given the constellation of clinical features, a clinical diagnosis of CIPA was made. Parents were advised to undergo nerve biopsy and genetic testing for confirmation, but these could not be performed due to financial limitations.

The primary goals of treatment were to prevent further self‐mutilation, manage complications, and improve the child's quality of life through supportive care. Protective gloves were fitted to minimize self‐inflicted injuries. Regular wound care with antiseptic dressings was initiated to promote the healing of ulcerative lesions on the fingers and lips. Iron supplementation was started to address the iron deficiency anemia. Nutritional rehabilitation, including a high‐calorie, high‐protein diet, was implemented to manage the child's severe malnutrition. A multidisciplinary approach involving the departments of pediatrics, ophthalmology, orthopedics, dentistry, and physiotherapy was adopted to address developmental delays, dental abnormalities, and corneal opacities.

## 4 Outcome and Follow‐Up

Parents were advised on managing febrile episodes and seizure prevention. Injury precautions were emphasized due to the absence of pain perception, with instructions to prevent accidental injuries, burns, and unnoticed infections. A regular follow‐up plan was established to monitor the child's condition.

## 5 Discussion

The clinical picture of our patient allows the diagnosis of CIPA. The patient exhibits impaired senses of pain and temperature, evidenced by repetitive self‐mutilating behaviors such as excessive biting of fingertips, lips, and tongue. These behaviors have resulted in significant tissue damage, including ulcerative lesions in the fingers, loss of tissue around the oral cavity, and deformation of the mandibular alveolar ridge. The dental findings reveal underdeveloped teeth in the upper jaw and a complete absence of teeth in the lower jaw, likely due to delayed eruption or self‐extraction associated with repetitive trauma. These manifestations align with known oral and dental complications in CIPA, where the lack of a pain‐mediated feedback mechanism leads to severe tongue and lip injuries, dental luxation, and auto‐extraction [11]. The presence of anhidrosis is inferred from the history of recurrent febrile episodes and convulsions, absence of sweating during fever, as well as dry and thick skin on the palms and soles. While autonomic function tests for sweating like iodine starch test or quantitative sudomotor axon reflex test could offer supplementary confirmation of anhidrosis, these were not pursued because the clinical diagnosis was unequivocal without additional testing, and results would not have altered our management approach. Additionally, the absence of corneal reflexes and corneal opacities are consistent with sensory impairment of this condition [9].

The differential diagnoses considered included Lesch–Nyhan syndrome, familial dysautonomia (FD), and anhidrotic ectodermal dysplasia. Lesch–Nyhan syndrome, an X‐linked disorder, also presents with self‐injurious behavior; however, it is characterized by hyperuricemia, dystonia, and spasticity [12], none of which were present in our patient. FD, which is also HSAN type III, typically presents with absent fungiform papillae on the tongue, diminished deep tendon reflexes, and alacrimia. Autonomic dysfunction in FD is more pronounced, with cardiovascular instability and severe gastrointestinal dysmotility [1]. Unlike CIPA, pain and temperature sensation in FD are only mildly to moderately reduced, and self‐mutilation is not a characteristic feature [1]. Anhidrotic ectodermal dysplasia was considered due to the absence of sweating, but it is primarily associated with distinct facial features, abnormally shaped or absent teeth, and widespread hypotrichosis [13], features not seen in our patient.

While the clinical presentation strongly supports the diagnosis of CIPA, confirmatory tests such as skin, nerve biopsy, and genetic analysis of the neurotrophic tyrosine kinase receptor 1 (NTRK1) gene provide definitive evidence [8]. Sweat glands on the skin biopsy appear normal [14], but electron microscopy reveals a lack of innervation of sweat glands with loss of unmyelinated sudomotor fibers, which could explain the anhidrosis [15]. Also, electron microscopy and morphometric analysis of the sural nerve show a marked reduction or absence of unmyelinated and small myelinated fibers responsible for pain and temperature sensation [16, 17]. Routine nerve conduction studies, such as electromyography, are often preserved [2, 18, 19].

CIPA is caused by autosomal recessive mutations in the NTRK1 gene, which encodes a receptor for nerve growth factor (NGF) [20]. The NTRK1 gene is located on chromosome 1q21‐22 [21]. The binding of NGF to NTRK1 is essential for the survival, growth, and function of nociceptive and autonomic neurons [8, 22]. Loss of NTRK1 function disrupts these processes, leading to the hallmark features of CIPA. Genetic testing to identify mutations in the NTRK1 gene is the gold standard for confirming the diagnosis but is cumbersome because of numerous mutations and polymorphisms and so is not routinely used for diagnosis [3, 19].

Although mutations in the *NTRK1* gene are classically associated with CIPA, recent studies have expanded the genotypic and phenotypic spectrum of this condition. The *SCN9A* gene, which encodes the Nav1.7 voltage‐gated sodium channel, plays a central role in nociceptive signal transmission. Loss‐of‐function mutations in *SCN9A* have traditionally been linked to congenital insensitivity to pain (CIP), where pain perception is absent but other autonomic functions, such as sweating, are typically preserved [23]. However, a recent case report has described individuals with *SCN9A* mutations presenting not only with CIP but also with anhidrosis [24]—features more characteristic of CIPA. These findings suggest that *SCN9A*‐related CIP may, in some instances, present with overlapping phenotypes and autonomic features, underscoring the genetic and clinical heterogeneity within the spectrum of hereditary pain disorders.

Although confirmatory tests such as skin and nerve biopsies or genetic analysis are definitive for diagnosing CIPA, their feasibility is limited in resource‐constrained settings like Nepal. Advanced diagnostic modalities such as nerve, skin biopsy, and genetic testing are unavailable in most healthcare centers. Even if these resources were available, the financial burden on patients would pose a significant barrier. Given these challenges, clinical diagnosis remains the most practical and accessible approach for identifying CIPA in Nepal. A careful assessment of the hallmark features—such as insensitivity to pain, anhidrosis, self‐mutilation, corneal opacities, and recurrent febrile episodes—enables timely recognition of the disorder. Early clinical diagnosis not only facilitates appropriate management and preventive care but also minimizes the risks of severe complications.

CIPA is a serious illness that may be fatal in the first years of life if hyperpyrexia is not properly corrected [2]. Management remains supportive, focusing on controlling hyperthermia, preventing self‐mutilation, and addressing dental and orthopedic complications. Early use of antipyretics, cool baths, and cold sponging is essential in preventing febrile seizures and life‐threatening hyperpyrexia. Protective measures such as padded clothing, routine skin inspections, and minimizing trauma help reduce the risk of unnoticed injuries. Non‐healing wounds require prompt care to prevent infection, mutilation, or sepsis. Orthopedic interventions may be needed for joint deformities and fractures, while dental care, including early tooth extraction and regular monitoring, helps prevent self‐inflicted oral injuries. Ophthalmologic evaluation is necessary to prevent corneal damage. A multidisciplinary team comprising pediatricians, neurologists, orthopedic specialists, dentists, and ophthalmologists plays a crucial role in optimizing outcomes and improving the quality of life for affected individuals.

## 6 Conclusion

CIPA is a rare and debilitating condition that poses significant diagnostic challenges, particularly in resource‐limited settings. While the diagnosis of CIPA can typically be made based on clinical features, confirmatory tests such as skin, nerve biopsy, and genetic analysis can provide definitive evidence. However, in resource‐limited settings like ours, the high cost and unavailability of these advanced diagnostic tools often necessitate proceeding with management based solely on clinical diagnosis. Early recognition is essential to prevent severe complications such as self‐inflicted injuries, infections, and hyperthermia. A multidisciplinary approach is crucial for optimizing patient care. Additionally, caregiver education on injury prevention and thermoregulation strategies plays a pivotal role in improving the quality of life for affected individuals. To the best of our knowledge, this is the first reported case of CIPA in Nepal.

## Author Contributions

**Sobin Pant:** conceptualization, methodology, resources, validation, visualization, writing – original draft, writing – review and editing. **Dibij Adhikari:** conceptualization, methodology, resources, validation, visualization, writing – original draft, writing – review and editing. **Prakash Pandey:** conceptualization, methodology, resources, validation, visualization, writing – original draft, writing – review and editing. **Binit Bajracharya:** conceptualization, methodology, resources, validation, visualization, writing – original draft, writing – review and editing. **Rizuna Sharma:** conceptualization, methodology, resources, supervision, validation, visualization, writing – original draft, writing – review and editing.

## Ethics Statement

The authors have nothing to report.

## Consent

Written informed consent was obtained from the patient's legal guardians for the publication of this case report and accompanying images in accordance with the journal's patient consent policy.

## Conflicts of Interest

The authors declare no conflicts of interest.

## Data Availability

Data supporting the findings of this case report are available from the corresponding author upon request.
